# The clinical significance of CD44v6 in malignant and benign primary bone tumors

**DOI:** 10.1186/s12891-023-06738-7

**Published:** 2023-07-25

**Authors:** Ameinh Hosseini, Amir Reza Eghtedari, Alireza Mirzaei, Pegah Babaheidarian, Samira Nekoufar, Narges Khademian, Khodamorad Jamshidi, Masoumeh Tavakoli-Yaraki

**Affiliations:** 1 Department of Biochemistry, School of Medicine, Iran University of Medical Sciences, P.O. Box: 1449614535, Tehran, Iran; 2 Bone and Joint Reconstruction Research Center, Shafa Orthopedic Hospital, Iran University of Medical Sciences, Tehran, Iran; 3 Department of Pathology, School of Medicine, Iran University of Medical Sciences, Tehran, Iran; 4 Department of Clinical Biochemistry, School of Medicine, Tarbiat Modares University, Tehran, Iran

**Keywords:** CD44v6, Osteosarcoma, Chondrosarcoma, Giant cell tumors, Malignancy

## Abstract

**Background:**

The objective of this study was to assess the expression profile of CD44v6, a potential cancer stem cell marker, and its diagnostic and predictive significance in three distinct types of primary bone tumors.

**Methods:**

In this study, we utilized real-time qRT-PCR and immunohistochemistry to examine the gene and protein levels of CD44v6 in a total of 138 fresh bone tissues. This included 69 tumor tissues comprising osteosarcoma (N = 23), chondrosarcoma (N = 23), and GCT (N = 23), as well as 69 corresponding non-cancerous tumor margins. Furthermore, we investigated the circulating level of CD44v6 by isolating peripheral blood mononuclear cells from 92 blood samples. Among these, 69 samples were obtained from patients diagnosed with primary bone tumors, while the remaining 23 samples were from healthy donors. The primary objectives of our investigation were to assess the correlation between CD44v6 expression levels and clinic-pathological features of the patients, as well as to evaluate the diagnostic and predictive values of CD44v6 in this context.

**Results:**

In patients with osteosarcoma and chondrosarcoma tumors, both the gene and protein expression of CD44v6 were found to be significantly higher compared to the GCT group. Furthermore, the circulating level of CD44v6 was notably elevated in patients diagnosed with osteosarcoma and chondrosarcoma in comparison to the GCT group and patients with malignant tumor characteristics. Additionally, we observed a strong correlation between the gene and protein levels of CD44v6 and important tumor indicators such as tumor grade, metastasis, recurrence, and size at the tumor site. CD44v6 shows potential in differentiating patients with bone tumors from both control groups and tumor groups with severe and invasive characteristics from those with non-severe features. Importantly, the expression level of CD44v6 also demonstrated predictive value for determining tumor grade and the likelihood of recurrence.

**Conclusion:**

CD44v6 is likely to play a role in the development of primary bone tumors and has the potential to serve as a diagnostic biomarker for bone cancer. However, to obtain more accurate and conclusive findings, further mechanistic investigations involving larger population samples are necessary.

## Introduction

Primary bone cancers are rare, heterogeneous sarcomas that originate from bones and are derived from mesenchymal cells [1]. These idiopathic tumors are often malignant and exhibit aggressive and invasive characteristics with diverse histopathological features that can affect various age groups. Their incidence rate is particularly notable among children and young adults [2]. The combination of chemotherapy and radiotherapy, along with tumor surgical resection, is the most important therapeutic strategy for patients, although its efficacy may be limited in some cases [3]. Consequently, efforts are being made to identify the signaling pathways and molecular mediators that play a role in the division, differentiation, and invasion of these cancer cells. The goal is to understand the etiology of these tumors and develop early diagnostic methods [4, 5]. The decisive role of the tumor microenvironment, extracellular matrix (ECM) mediators, and membrane receptors in tumor invasion and progression is well-described [6]. CD44 is a transmembrane glycoprotein the belongs to the cell adhesion molecules (CAMs) and interacts with ECM components and activate signaling pathways related to cell proliferation and growth [7]. The CD44 gene consists of 20 exons, and different isoforms are generated through complex alternative splicing of this gene. The ability of these isoforms to bind to ligands varies [8]. CD44 isoforms are present in actively dividing cells during embryonic development and can also be found in specific cancer types under certain circumstances, particularly in advanced stages. s [9, 10]. The intracellular, extracellular, and transmembrane domains of CD44 have the ability to bind to various ligands, including hyaluronic acid, collagen, osteopontin (OPN), integrin, and matrix metalloproteinases (MMPs) indicating the pivotal role of this protein in controlling cancer cell fate [11]. When cytoskeleton proteins bind to the cytoplasmic end of CD44, it leads to the connection between CD44 and the actin of the cytoskeleton. This interaction plays a role in regulating the transition from epithelial to mesenchymal states, as well as influencing cell motility, adhesion, and migration that rely on hyaluronic acid [12]. Interestingly, CD44 accounts as a cancer stem cell marker individually or in combination with other markers such as CD24, CD133 and CD34 [13]. The interaction between cancer stem cells (CSCs) and CD44 plays a crucial role in regulating various aspects such as cell survival, self-renewal, preservation, and resistance to chemotherapy. CD44 is essential for the adaptation of disseminated cancer cells to new environments and facilitates metastatic colonization [14, 15]. Studies have demonstrated that CD44-positive cancer cells in gastric cancer exhibit resistance to chemotherapy and radiation therapy. Furthermore, the suppression of CD44 cells resulted in reduced formation of spheroid colonies and tumor size in severe combined immune-deficient (SCID) mice, highlighting the tumorigenic potential of CD44 [16]. It is noteworthy that the capability of CD44 to bind with extracellular matrix components and hyaluronic acid allows cancer stem cells (CSCs) to receive and perceive signals and alterations in the tumor environment [10]. For instance, it was shown that binding of CD44 to hyaluronic acid and OPN resulted in triggering Nanog-Stat3 and c-Src kinase signaling pathway [17]. Given the significant involvement of CD44 in cancer cell growth, researchers have extensively studied its expression profile across various types of cancers. The data obtained consistently indicate an over-expression of CD44 in numerous tumor types [18, 19]. Due to the high expression of CD44, efforts have been made to investigate the diagnostic and prognostic value of CD44 and its isoforms in cancer [18, 20, 21]. While the expression profile of the CD44 standard isoform has been extensively investigated, there is still a gap in our understanding regarding the expression profile of CD44 functional variants and isoforms. Among them, CD44v6 stands out as an intriguing isoform due to its suggested diagnostic value as a marker for cancer stem cells (CSCs) in certain cancers, such as colorectal cancer [22]. Todaro et al. conducted a study revealing that CD44v6 is expressed by all colorectal cancer stem cells (CR-CSCs). The expression of CD44v6 is essential for the migration and formation of metastatic tumors, achieved through the activation of the WNT-β-catenin signaling pathway [23]. Propelling evidence recommends that CD44v6 compared to CD44 has a more specific expression in CSCs and may have a higher diagnostic value [24, 25], therefore, evaluating the expression profile and its changes in different tumors can provide new insights into early diagnosis of invasive tumors. In accordance, this study is aimed to determine the local (tumor site) and circulating (blood) expression level and diagnostic and predictive values of CD44v6 in three prevalent primary bone tumors (Osteosarcoma, Chondrosarcoma, and Giant cell tumors) with different tumor severity features.

## Materials and methods

### Sample preparation and collection

This study was approved ethically by the ethics committee of our institute and sample collection, procedures and informing the patients were conducted according to the declaration of Helsinki [26]. The total number of 138 fresh bone tissues including 69 tumor tissues (osteosarcoma [N = 23], chondrosarcoma [N = 23], Giant cell tumors (GCT) [N = 23]) and 69 non-cancerous tumor margins (osteosarcoma [N = 23], chondrosarcoma [N = 23], GCT [N = 23]) were collected for this survey. Also, the number of 92 blood samples including 69 samples from patients with primary bone tumors (osteosarcoma [N = 23], chondrosarcoma [N = 23], GCT [N = 23]) and 23 blood samples from healthy donors were enrolled. The patients underwent wide surgical resection at Shafa Orthopedic Hospital and tumor margins were taken away from a reliable distance from the cancerous tissue and both tumor and margin tissues were derived within 30 min following surgery and preserved based on our previously described procedure [27]. The blood sample was collected from each patient a few minutes before surgery as well as healthy donors who were matched to the patients as a matter of age and gender, then blood samples were subjected to peripheral blood mononuclear cell (PBMC) isolation. In fact, patients were entered the study before receiving drug treatment in order to reduce possible interactions. The tumor grade was determined due to the degree of differentiation and cellularity therefore low-grade tumors indicate well-differentiated and high-grade tumors indicate poor-differentiated tumors. In the current study both metastatic and non-metastatic as well as recurrent and non-recurrent tumors were included. Furthermore, by considering the patient’s pathology information, if the primary bone tumor of the individual had spread to remote organs, the local bone tumor was removed and it was classified as a metastatic tumor. Conversely, if no indications of distant metastases were observed in the patient, the tumor was categorized as non-metastatic. Additionally, a local recurrent bone tumor signifies the reappearance of the tumor one or more years after the patient has completed their initial treatment and tumor resection. In this regard, the medical history of the disease and previous treatment received were not considered. Instead, the focus was placed on the fact that the tumor was re-implanted in the individual and promptly operated upon and removed as soon as it was diagnosed. There were no cases reported of metastasis, tumor recurrence, or high-grade tumors among the GCT patients included in this study. . The patient’s demographic features are described in Table 1.

**Table 1:** The clinic- pathological features of patients with bone tumors

		Malignant bone tumor		Benign bone tumor
Demographic features	Groups	Osteosarcoma (N = 23)	Chondrosarcoma (N = 23)	GCT (N = 23)
Age	$<$30 30–60 60≤	6(26.1%) 13(56.52%) 4(17.39%)	0 19(82.60%) 4(17.39%)	12(52.17%) 10(43.47%) 1(4.34%)
Gender	Male Female	12(52.1%) 11(47.9%)	13(56.5%) 10(43.5%)	12(52.1%) 11(47.9%)
Tumor size (cm)	$<$10 $\ge$10	12(52.1%) 11(47.9%)	17(73.9%) 6(26.1%)	21(91.3%) 2(8.7%)
Tumor grade	Low (grade I/II) High (grade III)	7(30.4%) 16(69.6%)	13(56.5%0 10(43.5%)	23(100%) 0
Metastasis	Yes No	7(30.4%) 16(69.6%)	9(39.1%) 14(60.9%)	0 23(100%)
Tumor recurrence	Yes No	7(30.4%) 16(69.6%)	5(21.7%) 18(78.3%)	0 23(100%)

### Blood preparation

To evaluate the circulating level of CD44v6, the blood sample of each patient and healthy control were subjected to PBMC separation using Ficoll-Hypaque (Sigma Chemical Co, St Louis, MO, USA). The separation procedure is based on density gradient separation using several steps of centrifugation. Cells were washed and counted and an equal amount of cells were used for RNA extraction.

### Gene expression analysis

To evaluate CD44V6 gene expression, the Trizol (QIAGEN, USA) based on the phenol-chloroform protocol was applied as previously described [27]. The concentration and purity of extracted RNA were assessed by a Nanodrop spectrophotometer (Nanodrop Technologies). The optical absorption of samples at different wavelengths (260 and 280 nm) was measured and the ratio of OD260/OD280 = 2 indicates the purity of the extracted RNA due to protein contamination and the ratio OD260/OD230 = 2.2-2 indicates the purity of the extracted RNA due to contamination with buffer components. Moreover, electrophoresis (1% agarose gel) was used to evaluate the integrity of the extracted RNA. The PrimeScript First Strand cDNA Synthesis Kit (Takara, Japan) was applied to synthesize cDNA from extracted RNA (1 µg) and the SYBR Premix Ex Taq II (Takara, Japan) was used to evaluate CD44v6 gene level using Real-Time System (Applied Biosystems Step One Plus, USA). The CD44v6 (Target gene) and β-actin (House-keeping gene) primer sequences were as follows: CD44v6 forward primer: 5’–GGAACAGTGGTTTGGCAACA-3’, CD44v6 reverse primer: 5’-CTCTGCTGCGTTGTCATTGA − 3’ (Tm = 58), β-actin forward primer: 5’-GAT CTC CTT CTG CAT CCT GT-3’, β-actin reverse primer: 5’-TGG GCA TCC ACG AAA CTA C- 3’ (Tm = 57). The PCR reaction was set up as follows: Holding stage (95 °C, 10 min), Cycling stage (95 °C; 15 s. 56 °C; 25 s. 60 °C, 30 s for 40x), and melt curve stage (95 °C; 15 s. 65 °C, 35 s. 95 °C; 15 s). The melt curve of each primer and the proliferation plots were assessed to determine primer specificity for all samples. The CD44v6 gene expression level was calculated using the comparative Ct (2-ΔCt) method.

### Protein expression analysis

**Fig. 1:**
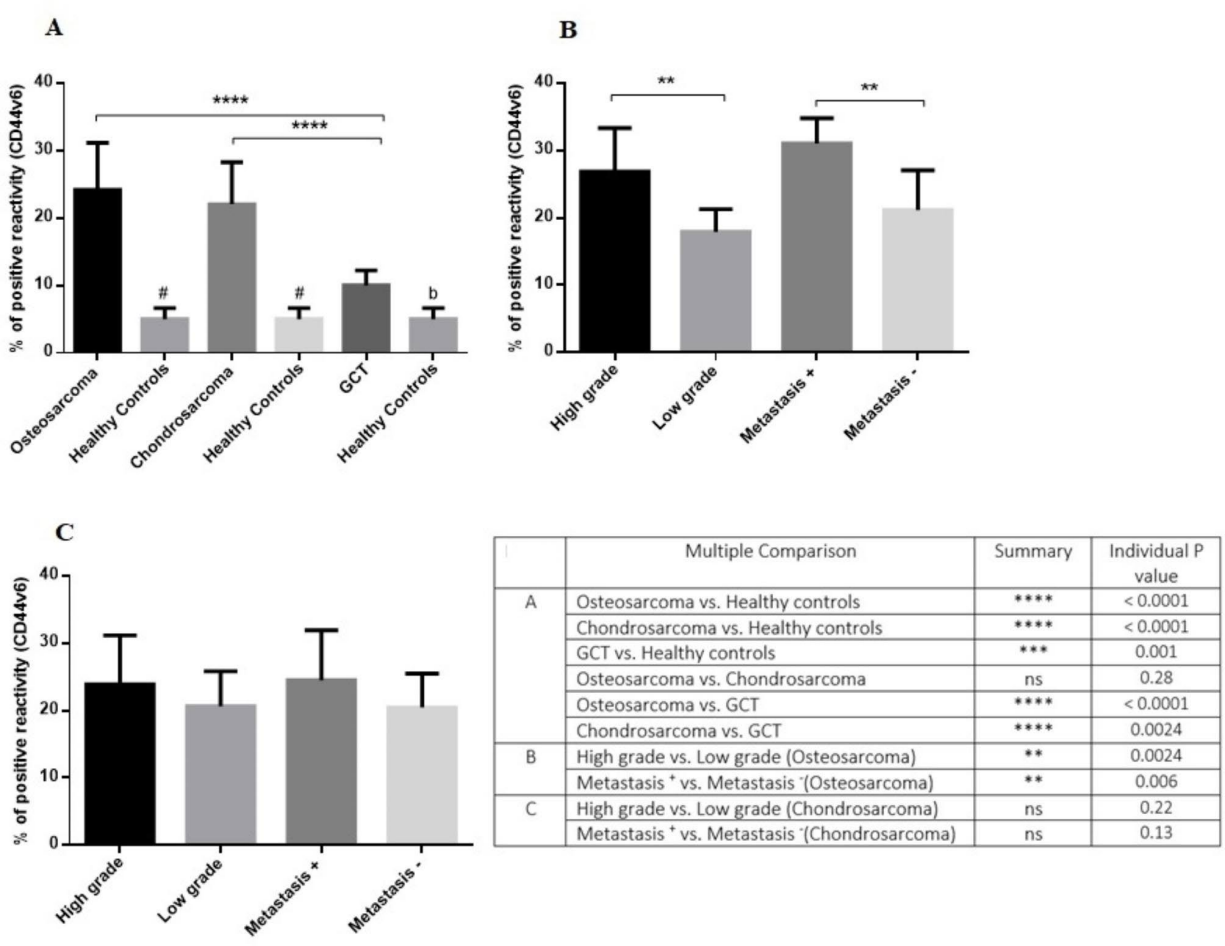
The protein level of CD44v6 in primary bone tumors. A higher protein level of CD44v6 was detected in osteosarcoma and chondrosarcoma tumors compared to GCT; while all three tumor types showed a significant expression difference compared to the corresponding healthy tissue **(A)**. The high and metastatic osteosarcoma tumors expressed higher CD44v6 levels compared to the low grade and non-metastatic tumors **(B)**; while chondrosarcoma tumors showed no remarkable difference in this regard **(C)**. The statistical differences between groups are shown as asterisks and the details of the compared groups and P-values are shown in the table (*= P < 0.05, **= P < 0.01, ****=P < 0.0001) also “ns” stands for not statistically significant

**Fig. 2:**
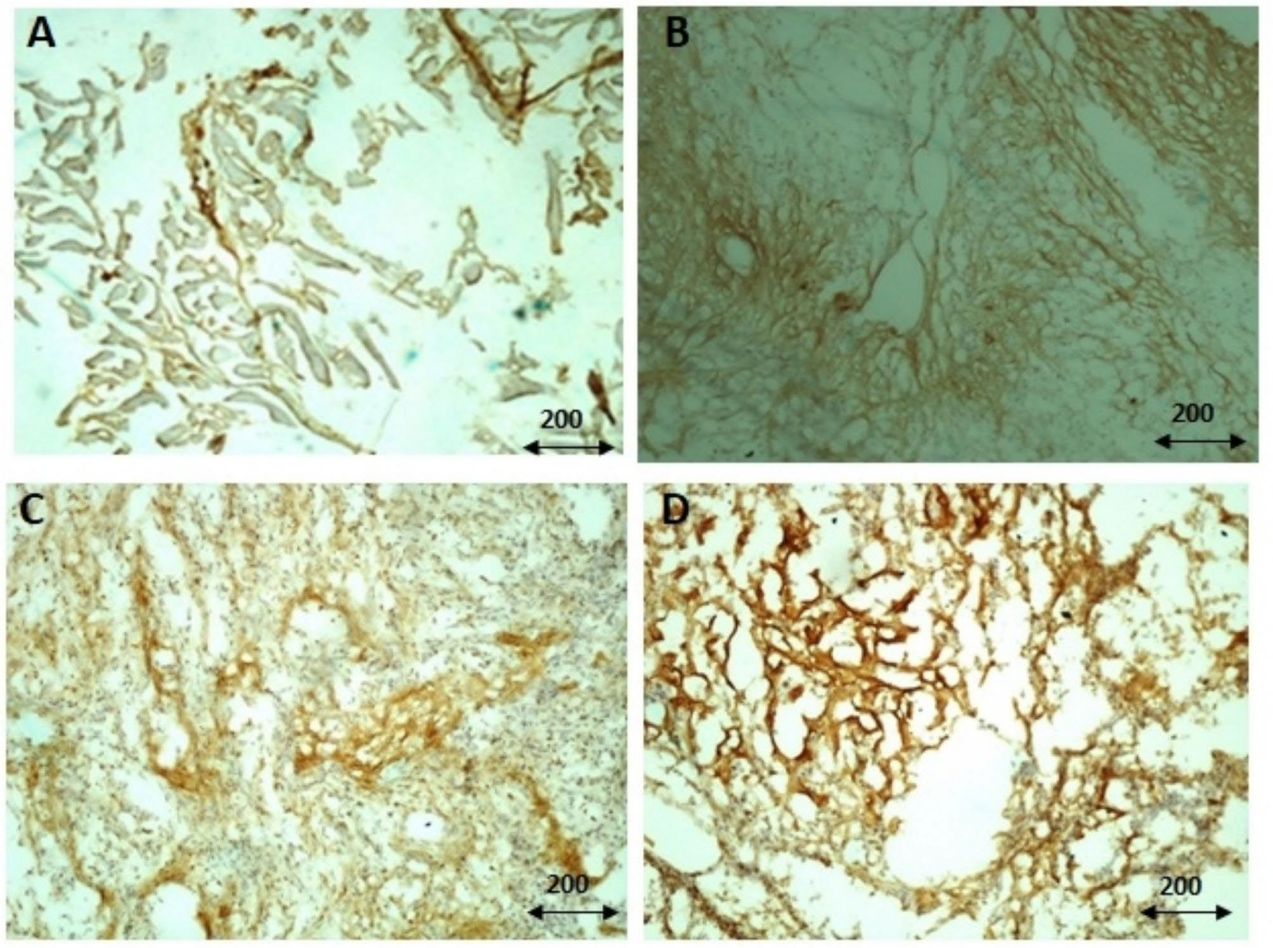
The immunohistochemistry staining of CD44v6 protein in primary bone tumors. The representative images of immunohistochemistry staining of CD44v6 protein are shown. **A:** represents negative immune-reactivity of CD44v6; **B:** represents the weak staining intensity of CD44v6 (< 10% immune reactivity); **C:** represents moderate intensity (10–20% immune reactivity) and **D:** represents strong intensity (> 20% immune reactivity). The scale of magnification is 200

Immunohistochemistry was performed to evaluate the protein level of CD44v6 in both tumor and margin samples. The anti-CD44v6 antibody (Catalog number: ab217902; Abcam) with the dilution of 1:500 and anti-mouse IgG HRP-conjugated secondary antibody (Catalog number: ab6728; Abcam) was applied for staining and detection. The detail of the protocol and analysis is based on the protocol described in our previous study [27, 28]. Briefly, tissue blocks were prepared in optimal cutting temperature (OCT) embedding medium and fixed in paraformaldehyde (4%). Tissue sections were prepared by cryotome and for inducing membrane permeability, Triton (3%) was used. The non-specific antigenic sites on tissue sections were blocked using goat serum (10%) and the intensity of staining was visualized using horseradish peroxidase activity and chromogens. The percentages of CD44v6 intensity for each sample were measured using Image J software [28] and presented as graphs (Fig. 1). The representative images for weak intensity (< 10% immune reactivity), moderate intensity (10–20% immune reactivity) and strong intensity (> 20% immune reactivity) is shown in Fig. 2.

### Statistical analysis

The Graph Pad Prism Version 6 (Graph Pad Software, San Diego California) and Statistical Package for Social Science (SPSS v.16) was used for statistical analysis, and statistically significant differences were indicated by P-value < 0.05. The normal distribution of data was evaluated using Kolmogorov-Smirnov analysis and differences between the expression levels of the CD44v6 gene and protein were analyzed using a parametric unpaired t-test. The correlation between CD44v6 gene and protein levels and the patient’s demographic features was calculated using Pearson’s correlation coefficient. The correlation coefficient (r) and the statistical differences between the compared groups were reported and are presented in Table 2. The logistic regression was used to determine the predictive values of variables in this study and the diagnostic values of the CD44v6 gene and protein in tumor and PBMC of patients with different types of bone tumors were assessed using the receiver operating characteristic (ROC) curve. For ROC analysis, the Youden index was considered to determine the area under the curve (AUC) and the optimal cut-off value points [29].

**Table 2:** The correlation of CD44v6 gene and protein levels in tumor and PBMC with different features of bone tumors

	PBMC	Tumor (Gene expression)	Tumor (Protein expression)
Variable	CD44v6	CD44v6	CD44v6
Age			
Correlation P value	0.012 0.924	0.339^**^ 0.005	0.300^*^ 0.013
Tumor size			
Correlation P value	0.288^*^ 0.017	0.647^**^ 0.000	0.507^**^ 0.000
Malignancy			
Correlation P value	0.468^**^ 0.000	0.543^**^ 0.000	0.745^**^ 0.000
Tumor grade			
Correlation P value	0.406^**^ 0.001	0.646^**^ 0.000	0.669^**^ 0.000
Metastasis			
Correlation P value	0.282^*^ 0.019	0.681^**^ 0.000	0.572^**^ 0.000
Recurrence			
Correlation P value	0.151 0.215	0.678^**^ 0.000	0.639^**^ 0.000
CD44v6 PBMC			
Correlation P value	1.000	0.296^*^ 0.014	0.309^**^ 0.010
CD44v6 gene			
Correlation P value	0.296^*^ 0.014	1.000	0.710^**^ 0.000
CD44v6 protein			
Correlation P value	0.309^**^ 0.010	0.710^**^ 0.000	1.000

## Results

### The gene expression level of CD44v6 in the tumor site and blood of patients with primary bone tumors

**Fig. 3:**
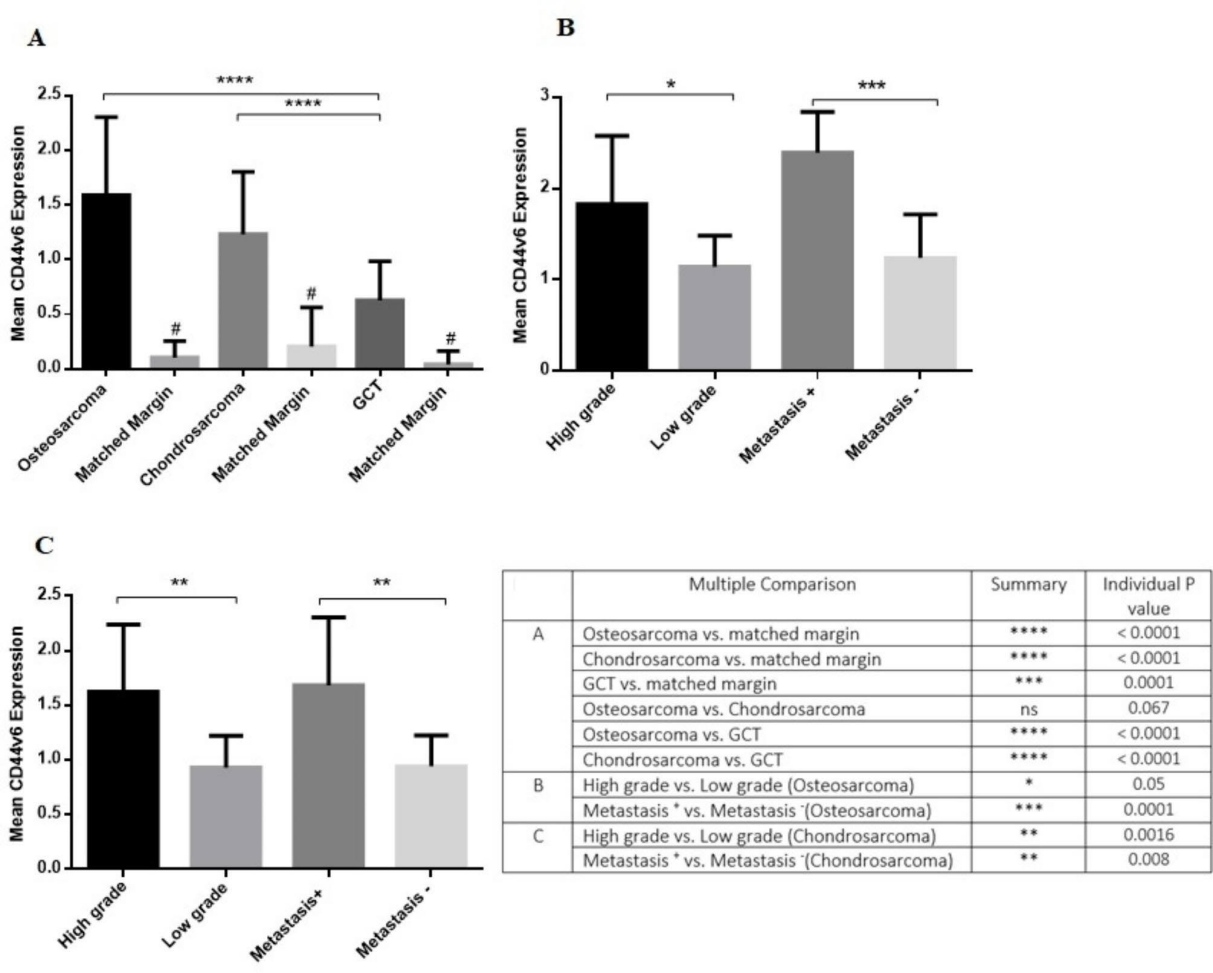
The gene expression level of CD44v6 in primary bone tumors. The gene expression levels of CD44v6 in osteosarcoma, chondrosarcoma and GCT compared to their matched control groups is shown **(A)**. The high grade and metastatic tumors expressed higher levels of CD44v6 compared to their counterparts in osteosarcoma **(B)** and chondrosarcoma **(C)** tumors. The statistical differences between groups are shown as asterisks and the details of the compared groups and P-values are shown in the table (*= P < 0.05, **= P < 0.01, ***= P < 0.001, ****=P < 0.0001), also “ns” stands for not statistically significant

**Fig. 4:**
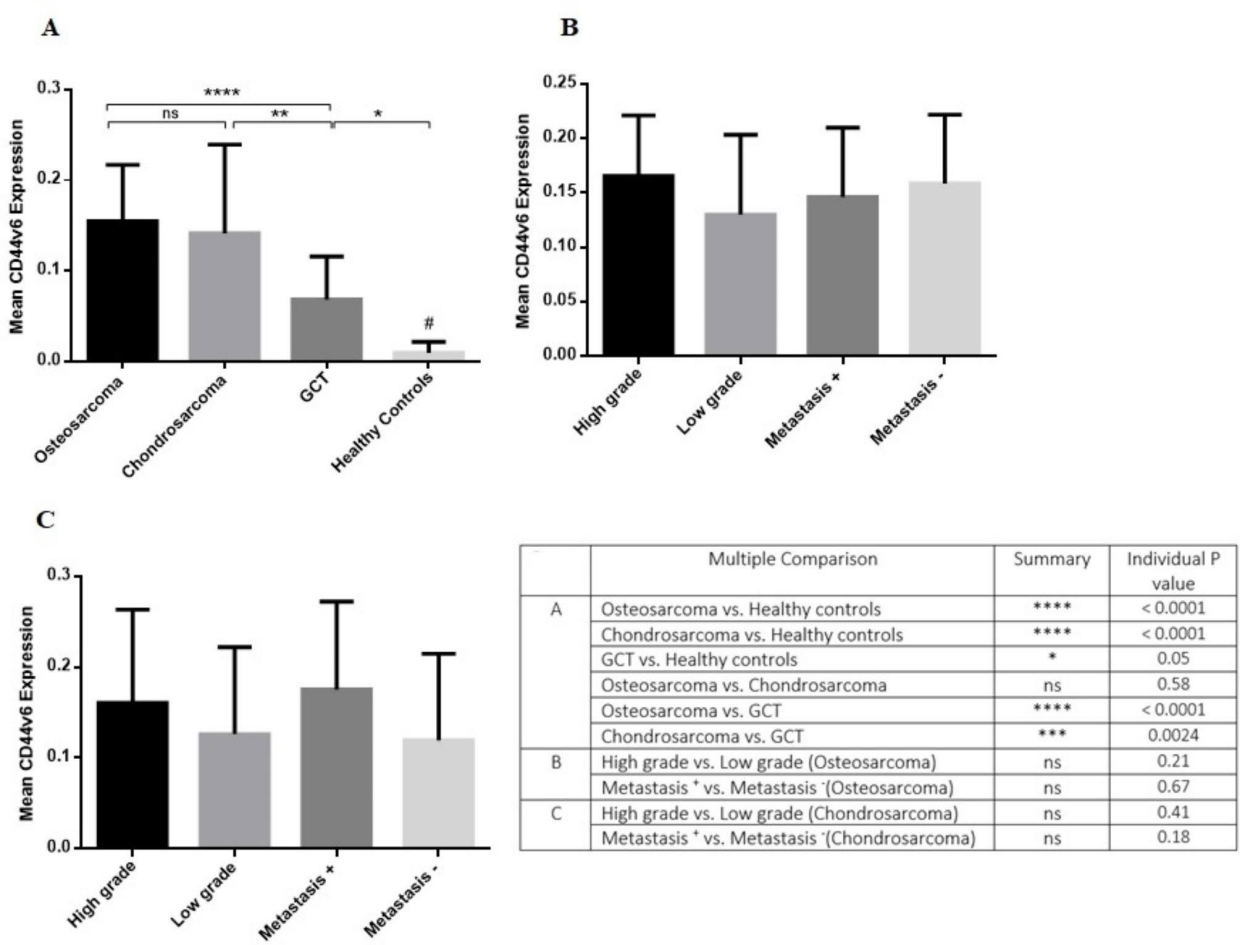
The gene expression level of CD44v6 in PBMCs of patients with primary bone tumors. The circulating level of CD44v6 in the PBMCs of patients with osteosarcoma and chondrosarcoma tumors was higher compared to patients with GCT; while the difference between osteosarcoma and chondrosarcoma groups was not significant **(A)**. No statistically significant difference was detected between the expression level of CD44v6 in the PMBCs of patients with osteosarcoma **(B)** and chondrosarcoma **(C)** as e matter of tumor grade and metastasis. The statistical differences between groups are shown as asterisks and the details of the compared groups and P-values are shown in the table (*= P < 0.05, **= P < 0.01, ****=P < 0.0001) also “ns” stands for not statistically significant

The expression level of CD44v6 increased significantly in osteosarcoma (P < 0.0001), chondrosarcoma (P < 0.0001) and GCT (P = 0.0001) groups compared to their matched non-cancerous tissues (Fig. 3A). Of notes, the mean and standard deviation (std) of CD44v6 in osteosarcoma, chondrosarcoma and GCT groups were 1.59 ± 0.71, 1.23 ± 0.57 and 0.62 ± 0.35, respectively. The CD44v6 level of expression was higher in osteosarcoma and chondrosarcoma tumors compared to the GCT group (P < 0.0001); while the difference between osteosarcoma and chondrosarcoma was not statistically significant (P = 0.067). Moreover, it was revealed that the CD44v6 expression level was increased in high grade (1.82 ± 0.74) compared to low grade (1.14 ± 0.34) osteosarcoma tumors (P = 0.05), also in metastatic (2.39 ± 0.44) versus non-metastatic (1.23 ± 0.47) osteosarcoma tumors (P = 0.0001) (Fig. 3B). The similar pattern of expression was detected regarding chondrosarcoma tumors with an increased level of CD44v6 in high grade (1.62 ± 0.61) compared to low grade (0.92 ± 0.29) (P = 0.001) and in metastatic (1.68 ± 0.62) compared to non-metastatic (0.94 ± 0.28) (P = 0.008) chondrosarcoma tumors (Fig. 3C). To investigate the expression pattern of CD44v6 in circulating cells of patients as a source of a non-invasive sample of patients, the PBMCs of patients were isolated and studied. Our data showed that the gene expression level of CD44v6 was higher in patients with osteosarcoma (0.15 ± 0.06) (P < 0.0001), chondrosarcoma (0.14 ± 0.09) (P < 0.0001) and GCT (0.068 ± 0.04) (P = 0.05) compared to healthy subjects (0.009 ± 0.01) (Fig. 4A). While the different between CD44v6 level of expression in osteosarcoma and chondrosarcoma groups was not statistically significant (P = 0.58); patients with osteosarcoma (P < 0.0001) and chondrosarcoma (P = 0.002) expressed higher level of CD44v6 in their blood compared to the patients with GCT (Fig. 4A). Comparing the expression level of CD44v6 in patients with osteosarcoma (Fig. 4B) and chondrosarcoma (Fig. 4C) did not show any significant difference in terms of grade and metastasis.

### The protein expression level of CD44v6 in different types of primary bone tumors

Based on our data, the protein level of CD44v6 increased significantly in osteosarcoma (P < 0.0001), chondrosarcoma (P < 0.0001) and GCT (P = 0.01) compared to their matched non-cancerous controls (Fig. 1A). A higher expression level of CD44v6 was detected in osteosarcoma (P < 0.0001) and chondrosarcoma (P = 0.0024) tumors compared to GCT; while the difference in the level of CD44v6 between osteosarcoma and chondrosarcoma was not remarkable (P = 0.28). A higher level of CD44v6 was detected in high grade (P = 0.0024) and metastatic (P = 0.006) osteosarcoma tumors compared to the low grade and non-cancerous tumors, respectively (Fig. 1B). However, the CD44v6 protein level showed no significant difference between different types of chondrosarcoma tumors (Fig. 1.C).

### The association of CD44v6 gene and protein expression levels with the patient’s demographic features and CD44v6 diagnostic and predictive values

**Fig. 5:**
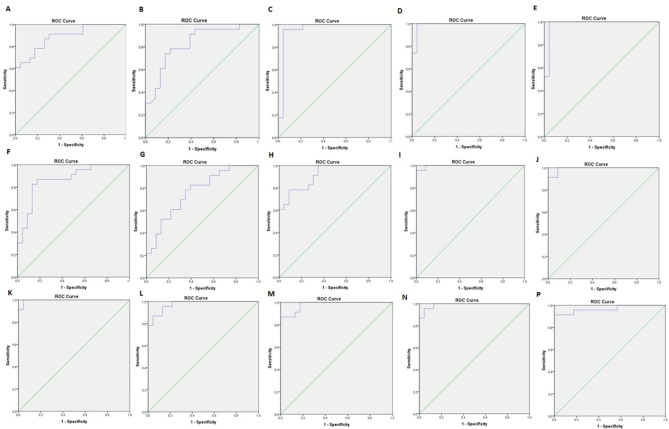
The ROC curve analysis. The diagnostic values of the CD44v6 gene and protein expression levels between different patient groups are calculated using ROC curve analysis. A-E: indicates ROC curve of CD44v6 gene level in tumor tissues between osteosarcoma and GCT **(A)**; between chondrosarcoma and GCT **(B)**; between GCT and non-cancerous tissues **(C)**, between osteosarcoma and non-cancerous tissues **(D)**; between chondrosarcoma and non-cancerous tissues **(E)**. F-J: indicates ROC curve of CD44v6 gene level in PBMCs of patients with osteosarcoma and GCT group **(F)**; chondrosarcoma and GCT **(G)**; GCT and non-cancerous tissues **(H)**, osteosarcoma and non-cancerous tissues **(I)**; chondrosarcoma and non-cancerous tissues **(J)**. K-P: indicates ROC curve of CD44v6 protein level in tumor tissues between osteosarcoma and GCT **(K)**; between chondrosarcoma and GCT **(L)**; between GCT and non-cancerous tissues **(M)**, between osteosarcoma tumors and non-cancerous tissues **(N)**; between chondrosarcoma and non-cancerous tissues **(P)**

Based on data that is shown in Table 2, the CD44v6 gene and protein expression level in the tumor site were significantly correlated with the patient’s age. Also, a significant correlation was detected between tumor size and the CD44v6 gene and protein level in tumor site and PBMCs of patients with primary bone tumors. Also, the correlation between the level of CD44v6 with tumor malignancy, grade, and metastasis was statistically significant in the tumor site and peripheral blood. A positive correlation was found between CD44v6 level of expression and tumor recurrence that was statistically significant while considering its expression level in the site of tumor not in the patient’s blood. Accordingly, a positive and significant correlation was found between the CD44v6 gene and protein level together and with the level of CD44v6 in the patient’s blood. As it is shown in Table 3, the CD44v6 protein level had a predictive value for tumor malignancy, tumor grade and recurrence, also CD44v6 gene expression level showed significant predictive values for tumor grade, metastasis and recurrence. The CD44v6 diagnostic values using ROC curve analysis showed that CD44v6 gene expression level in the tumor site can be of diagnostic value to distinguish between osteosarcoma and GCT (Cut off value < 0.83, AUC = 0.888, P = 0.0001), between chondrosarcoma and GCT (Cut off value < 0.8, AUC = 0.829, P = 0.0001), between GCT and non-cancerous bone tissues (Cut off value < 0.10, AUC = 0.959, P = 0.0001), between osteosarcoma tumors and non-cancerous bone tissues (Cut off value < 0.464, AUC = 0.989, P = 0.0001), and between chondrosarcoma and non-cancerous bone tissues (Cut off value < 0.497, AUC = 0.979, P = 0.0001).Notably, CD44v6 gene expression level in PBMCs of the patients had diagnostic value to discriminate between osteosarcoma and GCT (Cut off value < 0.10, AUC = 0.870, P = 0.0001), between chondrosarcoma and GCT (Cut off value < 0.085, AUC = 0.767, P = 0.002), between GCT and non-cancerous bone tissues (Cut off value < 0.017, AUC = 0.919, P = 0.0001), between osteosarcoma tumors and non-cancerous bone tissues (Cut off value < 0.028, AUC = 0.996, P = 0.0001), and between chondrosarcoma and non-cancerous bone tissues (Cut off value < 0.031, AUC = 0.992, P = 0.0001). Similarly, the CD44v6 protein level showed diagnostic values to distinguish between osteosarcoma and GCT (Cut off value < 14.03, AUC = 0.996, P = 0.0001), between chondrosarcoma and GCT (Cut off value < 12.64, AUC = 0.975, P = 0.0001), between GCT tumors and non-cancerous bone tissues (Cut off value < 7.3, AUC = 0.979, P = 0.0001), between osteosarcoma tumors and non-cancerous bone tissues (Cut off value < 10.69, AUC = 0.991, P = 0.0001), and between chondrosarcoma and non-cancerous bone tissues (Cut off value < 12.5, AUC = 0.968, P = 0.0001). The ROC analysis details are summarized in Table 4 and the curves are shown in Fig. 5.

**Table 3:** The Logistic regression of CD44v6 in tumor tissues

Dependent	Independent variable	OR	95% CI	P value
Malignancy (Benign Vs. Malignant)	CD44v6 (Gene)	0.38	0.001–107.5	0.74
	CD44v6 (Protein)	3.34	0.96–11.57	0.06
Tumor grade (Low grade Vs. High grade)	CD44v6 (Gene)	7.071	1.15–43.31	0.03
	CD44v6 (Protein)	1.206	1.05–1.39	0.01
Metastasis (Negative Vs. Positive)	CD44v6 (Gene)	14.602	2.18–97.94	0.006
	CD44v6 (Protein)	1.09	0.95–1.25	0.23
Recurrence (Negative Vs. Positive)	CD44v6 (Gene)	8.12	1.06–61.99	0.04
	CD44v6 (Protein)	1.26	1.01–1.57	0.04

**Table 4:** The diagnostic values of CD44v6 to differentiate different groups of primary bone tumors (ROC curve information)

Sample group	Sub-groups	Cutoff point	Sensitivity (%)	Specificity (%)	AUC	P-value
**CD44v6 gene in tumor**	Osteosarcoma Vs. GCT	< 0.83	87%	76%	0.888	0.0001
	Chondrosarcoma Vs. GCT	< 0.80	78%	70%	0.829	0.0001
	GCT Vs. Control	< 0.10	100%	96%	0.959	0.0001
	Osteosarcoma Vs. Control	< 0.464	100%	96%	0.989	0.0001
	Chondrosarcoma Vs. Control	< 0.497	95%	92%	0.979	0.0001
**CD44v6 gene in PBMC**	Osteosarcoma Vs. GCT	< 0.10	87%	83%	0.870	0.0001
	Chondrosarcoma Vs. GCT	< 0.085	69%	70%	0.767	0.002
	GCT Vs. Control	< 0.01	82%	78%	0.919	0.0001
	Osteosarcoma Vs. Control	< 0.028	100%	92%	0.996	0.0001
	Chondrosarcoma Vs. Control	< 0.031	95%	92%	0.992	0.0001
**CD44v6 protein in tumor**	Osteosarcoma Vs. GCT	< 14.03	100	96%	0.996	0.0001
	Chondrosarcoma Vs. GCT	< 12.43	91%	87%	0.975	0.0001
	GCT Vs. Control	< 7.3	91%	87%	0.979	0.0001
	Osteosarcoma Vs. Control	< 10.69	95%	96%	0.991	0.0001
	Chondrosarcoma Vs. Control	< 12.5	91%	87%	0.968	0.0001

## Discussion

Bone is a dynamic tissue capable of altering its cellular composition. It is rich in blood vessels, making it a favorable environment for tumor cell growth and a suitable substrate for tumor cell colonization. As a result, there is considerable interest in exploring the clinical relationship between various biomarkers and factors contributing to bone-related abnormalities [30, 31]. It is well-established that the survival and renewal of cancer stem cells (CSCs) are responsible for early diagnosis failures, inadequate response to chemotherapy, and inappropriate tumor resection [32]. Moreover, the fate of CSCs depends on their interactions with fibroblast cells, hematopoietic cells, and ECM compounds including hyaluronic acid [33, 34]. Conversely, CD44 and its variants, including CD44v6, serve as receptors for hyaluronic acid and are implicated in various processes such as cell adhesion, proliferation, differentiation, survival, and apoptosis of tumor cells. Their involvement contributes to the progression of tumors [19]. CD44v6 as CD44 isoform is expressed in epithelial cells as well as proliferating cells during embryonic development and in cancer cells [24, 25]. Our findings indicate a significant increase in the expression level of CD44v6 in primary bone tumors compared to their corresponding tumor margins. Although no difference was observed between osteosarcoma and chondrosarcoma, both groups exhibited significantly higher levels of CD44v6 compared to the GCT (giant cell tumor) group. Previously published data have revealed that CD44v6 over-expressed in tumor tissues of colorectal [35], breast [36], gastric [37], pancreas [38] and lung [39]. This overexpression has been correlated with cancer progression and poor prognosis. A meta-analysis study involving 3098 colorectal cancer patients demonstrated that CD44v6 serves as a superior prognostic biomarker for survival rate compared to CD44. Furthermore, CD44v6 expression was associated with increased severity of pathological grade and lower survival rates [40]. However, when it comes to bone sarcomas, the majority of studies have primarily examined CD44, with limited assessment of the CD44v6 variant, primarily in the context of osteosarcoma [19]. Accordingly, Deng et al. have shown that the expression of CD44v6 increased significantly in osteosarcoma tumors compared to osteochondromas which was associated with metastasis and a reduced survival rate [41]. Also, Kuryu et al. revealed that the CD44v6 protein level was increased in osteosarcoma tumors that was associated with decrease in survival rate and poor prognosis [42]. In our study, the observed lower expression level of CD44v6 in GCT (giant cell tumor) compared to osteosarcoma and chondrosarcoma tumors may be attributed to the non-aggressive nature of GCT samples [43] that were included in our study; however, no previously published data was found regarding the CD44v6 expression profile in GCT. As indicated previously [44], osteosarcoma and chondrosarcoma account for the most prevalent malignant bone tumors and our data showed a significant elevation of CD44v6 in osteosarcoma and chondrosarcoma tumors compared to their non-cancerous controls as well as GCTs. To support the notion of CD44v6’s role in tumor growth and invasion, experimental evidence has shown that the inhibition of CD44v6 using A5G27 polymer resulted in a reduction in cancer cell invasion and migration. This finding strongly suggests the involvement of CD44v6 in tumor invasion processes [45]. Moreover, the interaction of CD44v6 with ECM components stimulates PI3K/AKT pathway and triggers cell proliferation and mediates invasion [46]. Conversely, there is an interplay between CD44v6 and insulin-like growth factor 1 receptor (IGF-1R) that contributes to tumor cell homing and stimulates cell proliferation [47]. Additionally, CD44v6 is responsible for recruiting MMP-2 and MMP-9, thereby facilitating cell invasion and migration [48]. Consistent with this evidence, our findings revealed a significant increase in CD44v6 expression in high-grade and metastatic osteosarcoma and chondrosarcoma tumors. These results suggest the potential involvement of CD44v6 in the invasion and proliferation of bone tumor cells. However, further mechanistic studies are required to validate these findings. Additionally, in the current study, the level of CD44v6 expression was associated with tumor size and recurrence in osteosarcoma and chondrosarcoma tumors, highlighting its potential role in the division and reprogramming of bone tumor cells. Previous studies have primarily focused on investigating the protein level of CD44v6 through immunohistochemistry in osteosarcoma. However, in order to gain a comprehensive understanding of the changes in both the CD44v6 gene and protein levels in bone tumors, our current study simultaneously assessed the expression of the CD44v6 gene and protein. The data obtained revealed a consistent pattern of CD44v6 expression at both the gene and protein levels in osteosarcoma, chondrosarcoma, and GCT (giant cell tumor).While assessing biomarkers at the tumor site offers valuable insights into their status within the specific tumor population, liquid biopsy provides a distinct advantage by allowing non-invasive monitoring of tumor progression and response to therapies. This approach enables early detection and intervention before the disease worsens [49, 50]. Consistent with this, the circulating levels of CD44v6 in peripheral blood mononuclear cells (PBMCs) of patients was evaluated. Our data revealed an overexpression of CD44v6 in patients with osteosarcoma and chondrosarcoma compared to patients with GCT (giant cell tumor). However, no significant association was found between the circulating level of CD44v6 and tumor grade or metastasis. No study regarding the level of CD44v6 in PBMCs of patients with bone sarcoma had been conducted so far and our study offers the advantage of concurrently assessing the alterations in CD44v6 profile in both liquid biopsy and the tumor site. Synder et al. provided evidence that CD44v6-positive breast tumor cells exhibit stem cell-like properties, including self-renewal and tumorigenesis. This suggests the diagnostic significance of assessing CD44v6 in circulating tumor cells (CTCs) [24]. Zhou Gang et al. demonstrated that mRNA levels of CD44v6 in peripheral blood mononuclear cell (PBMC) samples from patients with pancreatic cancer were elevated compared to the control group. Furthermore, the levels of CD44v6 gene and protein expression were found to be associated with tumor stage, tumor differentiation, and liver metastasis [51]. Previous studies have suggested the potential use of peripheral blood mononuclear cells (PBMCs) as a source of circulating tumor cells (CTCs) [52]. However, the presence of non-tumor cells within these populations can impact the accuracy of gene status assessment. Therefore, it is necessary to evaluate the expression profile of Cd44v6 in isolated and characterized CTCs in order to reach a definitive conclusion. Additionally, expanding the study to include a broader range of disease severity and patient population would further enhance its scope and relevance. It is important to highlight that this study had a few limitations. First, in this study, ethical constraints led to the consideration of the tissue surrounding the tumor as a control tissue that is deemed healthy. Although this approach is commonly employed in various studies [53, 54], it is preferable to utilize healthy tissue with the same cellular origin as the tumor for improved accuracy and reliability. Second, while this study offers the benefit of comparing the CD44v6 profile alterations in three distinct bone tumor types, it is also recommended to investigate the expression pattern changes of this factor within both benign and malignant tumors for each of these tumor types. To arrive at a more accurate inference regarding the significance of the CD44v6 biomarker’s role, it becomes imperative to analyze the impact of treatment protocols and chemotherapy while assessing the fluctuating trends of this element in patients subjected to various chemotherapy regimens. Additionally, for a more comprehensive exploration of recurrent tumors and their distinctions, it is advisable to simultaneously investigate both the primary tumor and the relapsed tumor of each patient.

## Conclusion

According to our data, the levels of gene and protein expression of CD44v6 were found to be higher in osteosarcoma, chondrosarcoma, and GCT tumor tissues compared to normal bone tissue. Additionally, the increased level of CD44v6 was significantly associated with tumor grade, metastasis, and the extent of tumor deterioration. These findings indicate that it is essential to investigate the potential biomarker role of CD44v6 individually for each tumor type due to variations in gene expression. Furthermore, we observed a simultaneous increase in CD44v6 expression and a similar pattern of expression both at the tumor site and in peripheral blood samples of patients. This suggests that evaluating CD44v6 expression in the tumor site and blood of primary bone tumors can serve as informative measures for monitoring disease progression over time. Our study, along with other research, provides evidence supporting the potential prognostic value of CD44v6 in primary bone tumors for further evaluations.

## Data Availability

All data regarding this study are included in this published paper.

## Abbreviations

ECM: Extra cellular matrix
CAMs: Cell adhesion molecules
OPN: Osteopontin
MMP: Matrix metalloproteinases
CSC: Cancer stem cells
SCID: Severe combined immune-deficient
CR-CSC: Colorectal cancer stem cells
GCT: Giant cell tumor
PBMC: Peripheral blood mononuclear cell
OCT: Optimal cutting temperature
ROC: Receiver operating characteristic
AUC: Area under the curve
std: Standard deviation
IGF-1R: Insulin-like growth factor 1 receptor
CTC: Circulating tumor cells
